# Prophylactic clip application for large pedunculated polyps before snare polypectomy may decrease immediate postpolypectomy bleeding

**DOI:** 10.1186/s12876-020-01210-5

**Published:** 2020-03-12

**Authors:** Jae Seung Soh, Myeongsook Seo, Kyung-Jo Kim

**Affiliations:** 1 Division of Gastroenterology, Department of Internal Medicine, Hallym University Sacred Heart Hospital, University of Hallym College of Medicine, Anyang, Republic of Korea; 2 Department of Internal Medicine, Gangneung Asan Hospital, University of Ulsan College of Medicine, Gangneung, Republic of Korea; 3 Department of Gastroenterology, Asan Medical Center, University of Ulsan College of Medicine, 88 Olympic-ro 43-gil, Songpa-gu, Seoul, 05505 Republic of Korea

**Keywords:** Post-polypectomy bleeding, Clip, Pedunculated polyps

## Abstract

**Background:**

Although prophylactic clip application before polypectomy may prevent postpolypectomy bleeding (PPB), the usefulness of prophylactic clipping in the treatment of large pedunculated polyps is controversial in some prospective randomized studies. This study was conducted to evaluate the efficacy of prophylactic clip application and to investigate the predictors of PPB in large pedunculated colorectal polyps.

**Methods:**

A total of 137 pedunculated polyps (size ≥1 cm) in 116 patients were prospectively included and randomized into group A (with clipping) and group B (without clipping), and resected. The occurrences of immediate PPB (graded 1–4) and delayed PPB were compared.

**Results:**

Sixty-seven polyps were allocated in group A and 70 polyps in group B. In both groups, the median polyp diameter was 15 mm (*P* = 0.173) and the median stalk diameter was 3 mm (*P* = 0.362). Twenty-eight (20.4%) immediate PPB episodes in 137 polyps occurred, 6 (9.0%) in group A and 22 (31.4%) in group B (*P* = 0.001). However, the occurrence of delayed PPB was not different between the groups (*P* = 0.943). Prophylactic clip application decreased the occurrence of immediate PPB (odds ratio 0.215, 95% confidence interval 0.081–0.571). Moreover, polyp size ≥20 mm and stalk diameter ≥ 4 mm increased the risk of immediate PPB.

**Conclusions:**

Clip application before polypectomy of ≥1 cm pedunculated polyps is effective in decreasing the occurrence of immediate PPB. Thus, clip application should be considered before performing snare polypectomy, especially for large polyps with a thick stalk.

**Trial registration:**

This research was studied a prospective maneuver and enrolled in a registry of clinical trials run by United States National Library of Medicine at the National Institutes of Health (ClinicalTrials.gov Protocol Registration and Results system ID: NCT01437631). This study was registered on September 19, 2011.

## Background

Colorectal cancer (CRC) is a major cause of cancer-related death worldwide [1]. Colonoscopic removal of neoplastic polyps has been shown to reduce the incidence of both CRC and CRC-associated mortality [2, 3]. Although most of colorectal polyps are managed with endoscopic resection, adverse events such as bleeding, perforation, and infection can occur after colonoscopic polypectomy [4].

Of the polypectomy-associated adverse events, postpolypectomy bleeding (PPB) is the most common, and the rate of PPB was reported to be approximately 0.3–6.1% in previous studies [5–7]. The causes of PPB consist of patient-related factors including age, underlying disease, and prior use of antiplatelets or anticoagulants; polyp-related characteristics including type, size, and location; and procedure-related factors including type of electrosurgical current and submucosal injection of epinephrine-containing solution [8–10]. Among these factors, a polyp ≥1 cm in size is the biggest risk factor for PPB [10].

Injection of epinephrine-saline into the stalk may be performed as a prophylactic maneuver to reduce immediate PPB [11]. However, its effect might be temporary. A standard detachable snare has been used for the removal of large pedunculated polyps [12]. The detachable snare could obtain optimal tightness at the stalk of the polyp, which may be sufficient to prevent PPB; however, placing a detachable snare is technically difficult, and polyp entrapment, pedicle resection from overtightening, or slippage of the snare could occur. A clipping method has been used to control and prevent bleeding during colonoscopic polypectomy since the early stage of polypectomy [13]. Theoretically, prophylactic clip application may prevent PPB [14, 15]. However, a prospective randomized controlled trial failed to prove the benefits of prophylactic clipping in patients with large pedunculated polyps [15]. In addition, prophylactic use of clips in the removal of large pedunculated polyps leads to a further risk of mucosal burn and perforation, and cannot reduce the risk of PPB.

This prospective randomized study was conducted to evaluate the usefulness of prophylactic clip application in preventing PPB after colonoscopic polypectomy in patients with large pedunculated polyps (size ≥1 cm), and to investigate the predictors associated with PPB.

## Methods

### Sample size estimation

The sample size was calculated with the assumption that prophylactic clip application would reduce the bleeding rate by 15%, compared with non-prophylactic therapy, based on 5% of the PPB rate. Given α = 0.05 and a power of 80%, we required a sample size of 192 patients.

### Patients

The local ethics committee at Asan Medical Center approved the use of clinical data for this study (Number of institutional review board 2011–000653). Informed consent was obtained from every enrolled patient before each procedure. We recruited patients who were scheduled to undergo colonoscopy or colonoscopic polypectomy at Asan Medical Center in Seoul, Korea, from September 2011 to April 2015. All patients underwent complete colonoscopy with cecal intubation and snare polypectomy for pedunculated colon polyps. Pedunculated polyps ≥1 cm were included in the present study. We excluded patients who met the following criteria: (i) age < 20 years; (ii) polyps of lateral spreading tumor or sessile type without an identified stalk; (iii) abnormal coagulogram (platelet count, prothrombin time, and partial thromboplastin time); (iv) inflammatory bowel disease, chronic renal failure, or chronic liver disease; and (v) use of anticoagulant medication such as warfarin. We have obtained informed consents from 119 patients; however, 3 patients did not undergo polypectomy by personal reason and were excluded from the study. A total of 137 large pedunculated polyps in 116 patients were finally included. We enrolled only the patients with pedunculated polyps, and excluded those having both pedunculated polyp and sessile polyp or lateral spreading tumor as showed in the exclusion criteria from the baseline. All pedunculated polyps were randomized using a computerized random number generator, and removed according to the randomized treatment assignment, as follows: (i) snare polypectomy after prophylactic clip application on the stalk of the polyp (group A) and (ii) snare polypectomy without prophylactic clip application (group B) (ClinicalTrials.gov Protocol Registration and Results system ID: NCT01437631). Eighteen patients had multiple pedunculated polyps; 16 patients with 2 pedunculated polyps, one with 3 pedunculated polyps, and one with 4 pedunculated polyps. The polyps were randomized along the same method. The patients’ demographics including age, sex, weight, and height; underlying diseases such as hypertension, diabetes mellitus, and cardiovascular disease; and medical history use of antiplatelet agents was obtained through an interview. Body mass index was calculated as the weight in kilograms divided by the height in meters squared. Cardiovascular diseases included well-controlled coronary diseases such as myocardial infarction and angina, and heart failure. Twenty-one patients (17.9%) took antiplatelet medications such as aspirin, clopidogrel, or beroprast. The patients were instructed to discontinue taking these medications 5–7 days before the procedure and to resume taking the medications the following day after the procedure.

### Colonoscopy procedures

Bowel preparation was performed with 4 L polyethylene glycol-electrolyte solution or 2 L polyethylene glycol-electrolyte solution containing ascorbic acid. The Aronchick scale was used to evaluate bowel preparation status [16]. Patients with poor preparation, including the presence of semi-solid stool that could not be suctioned or washed away and visualization of < 90% of the surface, were excluded from the study or subjected to repeated bowel cleansing until adequate preparation was achieved.

**Fig. 1:**
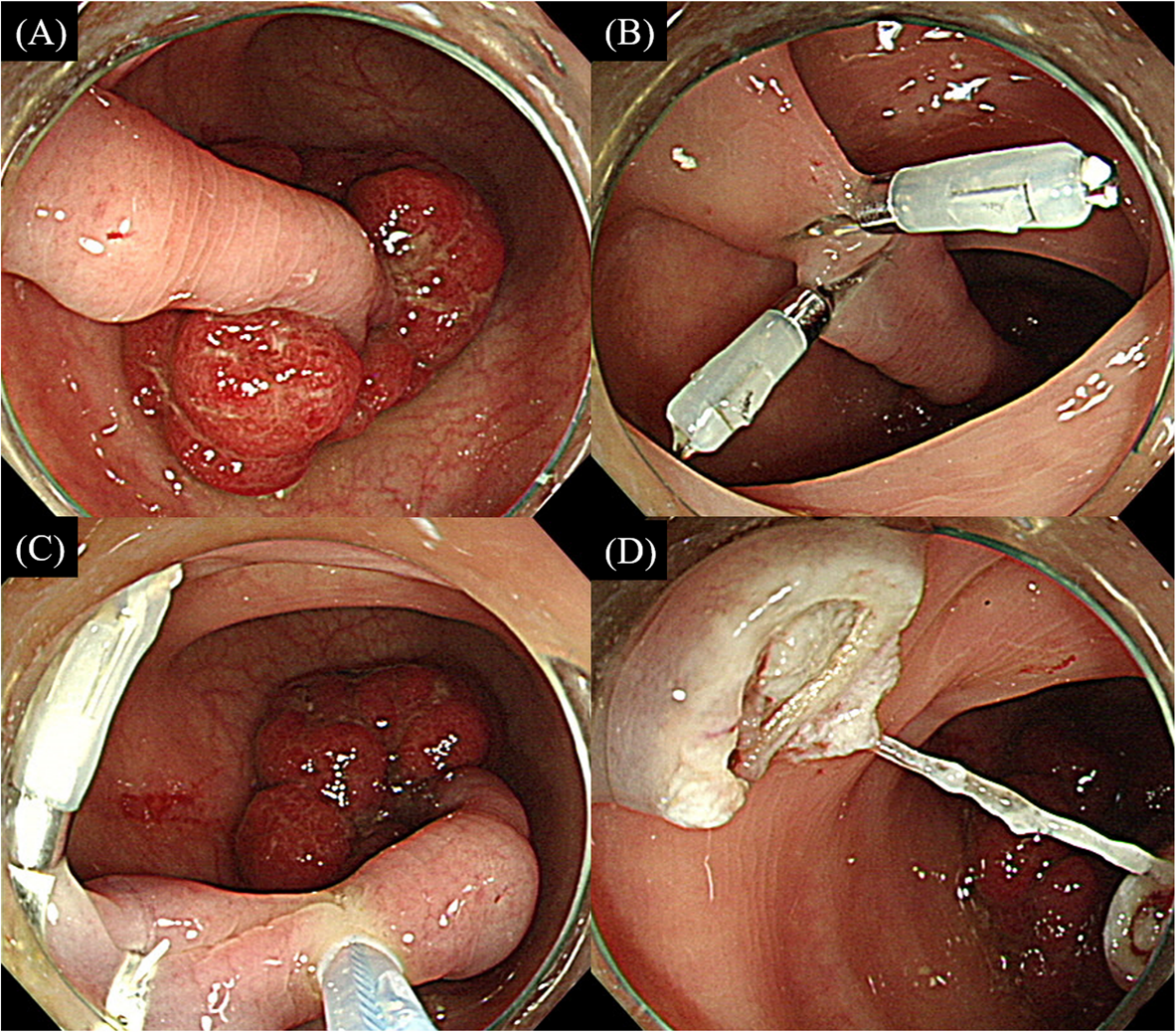
A case of prophylactic clip application for a large pedunculated polyp. **a** A 2.5-cm pedunculated polyp with a 4-mm-diameter stalk was noted in the sigmoid colon. **b** Two clips were placed on the lower part of the stalk for prophylaxis. **c** Snare polypectomy was performed on the stalk just above the clips. **d** There was no evidence of bleeding on the resected site

Procedures were performed under conscious sedation with intravenous midazolam only if requested by the patients. Colonoscopic polypectomy was performed using standard colonoscopes (CF-Q260I or H260I; Olympus Optical, Tokyo, Japan). All the polypectomies were performed by a single endoscopist (KJK) with ≥10 years’ experiences in therapeutic colonoscopy to minimize technical variability under the same techniques and equipment setting. All the patients were monitored with pulse oximetry during the procedure. The size of polyps was measured by comparing it with the size of the biopsy forceps, and the stalk diameter of the polyps was measured using the scale of the snare. The location of the polyps was divided into proximal (from the cecum to the transverse colon) and distal (from the splenic flexure to the rectum). All included pedunculated polyps were resected using a standard snare without diluted epinephrine-saline injection. Only for group A, before polypectomy, 1–3 hemoclips (HX-610-090 L, Olympus) were placed on the stalk of the polyp according to the gross stalk diameter by a single experienced endoscopist (Fig. 1). When the stalk was not covered with the first clip adequately, a second one was applied to the opposite side, or more clips were used until most of the pedicle was covered fully. An electrosurgical unit (VIO 300D; ERBE, Tübingen, Germany) was always set according to the manufacturer’s instructions: Endocut Q mode (effect 3, cut duration 2, and cut interval 6) and forced coagulation (effect 2, 40 W). All the polyps were resected with the same method, using sequential application with forced coagulation and Endocut Q mode in both groups. A single continuous application of electrosurgical current was attempted until the polypectomy was completed. The resected polyps were sent for histopathological examination and classified according to the standard protocol by the World Health Organization [17].

### Post-Polypectomy bleeding

Immediate PPB was defined as continuous bleeding for ≥30 s at the polypectomy site and graded from 1 to 4, according to a published study that evaluated the risk factors for PPB [8]. Grade 1 was defined as spontaneous hemostasis within 60 s second. Grade 2 was defined as continuous but decreased oozing over 60 s second. Grade 3 was defined as continuous oozing over 60 s second that needed endoscopic treatment. Grade 4 was defined as active spurting that needed endoscopic treatment. In cases in which immediate PPB occurred, additional clips or an endoloop (Polyloop, Olympus) was added to control bleeding. All cases of immediate PPB were controlled successfully without complications in both groups.

Delayed PPB was defined as hematochezia from the day of the procedure to the day of the first visit at the outpatient clinic, and categorized into significant or minor bleeding. Significant bleeding was defined as massive hematochezia and/or hemoglobin loss of > 2 g/dL that required endoscopic hemostasis. Minor bleeding was defined as self-limited hematochezia and hemoglobin loss of < 2 g/dL that did not require endoscopic hemostasis. All the enrolled patients visited outpatient clinic after polypectomy as scheduled between 14 and 21 days, and were asked about a history of hematochezia. Patients were instructed to visit the emergency room or call our endoscopic unit if they experience gross hematochezia or dizziness.

We compared the hospital stay between the two groups to evaluate whether immediate or delayed PPB lengthened hospitalization or not.

### Statistical analyses

The chi-square test or 2-tailed Fisher’s exact test was used to evaluate the association among various categorical variables, and the independent-sample *t*-test was used for non-categorical variables. Cox regression analysis was performed to examine the predictors associated with PPB. The Statistical Package for the Social Sciences (SPSS) version 21.0 (SPSS Inc., Chicago, IL, USA) was used for all statistical analyses. A *P*-value of < 0.05 was considered statistically significant.

## Results

### Comparison of the characteristics of patients, polyps, and procedures between groups a and B

Of the 137 polyps in 116 patients enrolled in the present study, 67 polyps were allocated to group A and the remaining 70 polyps to group B. Eighteen patients had more than 2 polyps (16 patients with 2 polyps, 1 patient with 3 polyps, and 1 patient with 4 polyps). All the patients completed follow-up visit. Table 1 lists the characteristics of the patients, polyps, and procedures in groups A and B. The median age of the patients in groups A and B was 57 years (range, 31–75 years) and 59 years (range, 33–78 years), respectively (*P* = 0.939). Sex; comorbid disease such as hypertension, diabetes, cardiovascular disease; use of antiplatelet medication; and body mass index were not different between the groups. The laboratory findings, including hematocrit, platelet count, prothrombin time, and partial thromboplastin time, were not different between the groups, as shown in Table 1. Bowel preparation was excellent or good in 76.1% of group A and 84.3% of group B patients (*P* = 0.329). The median size and stalk diameter of the polyps were 15 mm and 3 mm, respectively, in both groups. There was no difference in the location of the polyps (*P* = 0.366). Excluding bleeding, other complications including mucosal burn and perforation were not occurred in all cases with clipping.

**Table 1:** Baseline characteristics of patients, polyps, and procedures in groups A and B

Patient-related factors	Group A (*n* = 53)	Group B (*n* = 63)	*P*-value
Age, median, years (range)	57 (31–75)	59 (33–78)	0.939
Male sex, no. (%)	40 (75.5)	50 (79.4)	0.659
HTN, no. (%)	15 (28.3)	27 (42.9)	0.123
DM, no. (%)	4 (7.5)	7 (11.1)	0.752
Cardiovascular disease, no. (%)	1 (1.9)	4 (6.3)	0.374
Use of antiplatelet, no. (%)	7 (13.2)	13 (20.6)	0.332
BMI, median, kg/m^2^ (range)	23.6 (14.1–34.0)	24.2 (16.5–31.3)	0.455
Laboratory findings			
Hematocrit, mean, % (SD)	41.8 (4.6)	41.6 (4.5)	0.812
Platelet count, mean, × 10^3^/μL (SD)	251 (64)	243 (66)	0.519
Prothrombin time, mean, INR (SD)	0.98 (0.08)	0.98 (0.06)	0.962
PTT, mean, s (SD)	28.7 (2.5)	27.8 (2.2)	0.053
Polyp-related factors	Group A (*n* = 67)	Group B (*n* = 70)	*P*-value
Polyp size, median, mm (range)	15 (10–30)	15 (10–40)	0.173
Stalk diameter, median, mm (range)	3 (1–5)	3 (1–10)	0.362
Location of polyp			0.366
Right colon, no. (%)	28 (41.8)	24 (34.3)	
Left colon, no. (%)	39 (58.2)	46 (65.7)	
Histology			0.344
Adenoma, low grade, no. (%)	46 (68.7)	50 (71.4)	
Adenoma, high grade, no. (%)	8 (11.9)	9 (12.9)	
Adenocarcinoma, no. (%)	3 (4.5)	4 (5.7)	
Traditional serrated adenoma, no. (%)	3 (4.5)	4 (5.7)	
Hamartomatous polyp, no. (%)	3 (4.5)	1 (1.4)	
Inflammatory polyp, no. (%)	2 (3.0)	1 (1.4)	
Filiform serrated adenoma, no. (%)	1 (1.5)	1 (1.4)	
Hyperplastic polyp, no. (%)	1 (1.5)	0 (0.0)	
Procedure-related factors	Group A (n = 53)	Group B (n = 63)	*P*-value
Bowel preparation			0.329
Excellent, no. (%)	15 (28.3)	25 (39.7)	
Good, no. (%)	32 (60.4)	31 (49.2)	
Adequate, no. (%)	6 (11.3)	7 (11.1)	

HTN, hypertension; DM, diabetes mellitus; BMI, body mass index; SD, standard deviation; INR, international normalized ratio; PTT, partial thromboplastin time

### Comparison of the rates of PPB between groups a and B

**Fig. 2:**
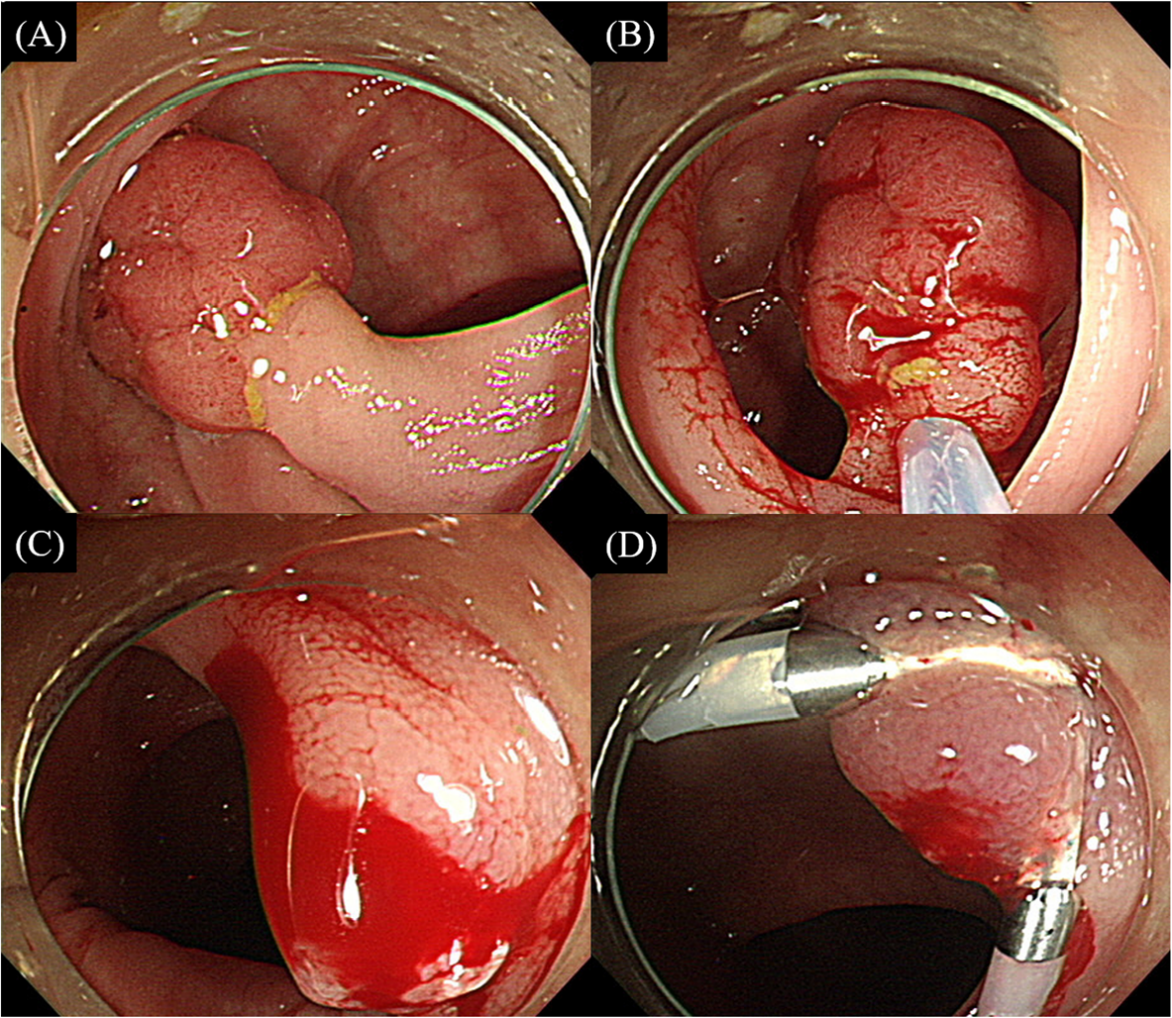
A case of postpolypectomy bleeding after the removal of a large pedunculated polyp. **a** A 2-cm pedunculated polyp with a 4-mm-diameter stalk was found in the sigmoid colon. **b** Snare polypectomy was performed. **c** Spurting bleeding occurred from the resected site of the stalk. **d** The bleeding ceased after the application of 2 clips on the bleeding site

Immediate PPB occurred in 6 polyps (9.0%) in group A and in 22 polyps (31.4%) in group B, which was significant (*P* = 0.001) (Table 2). In addition, immediate PPB of grades 3 and 4 that required endoscopic treatment occurred more frequently in group B (*P* = 0.008); however, the rates of grade 1 and 2 PPB were not different (*P* = 0.208). Figure 2 presents a case in group B that showed spurting bleeding after polypectomy. The bleeding ceased after the application of 2 hemoclips on the bleeding site.

**Table 2:** Comparison of immediate and delayed postpolypectomy bleeding rate between groups A and B

Variables	Group A (n = 67)	Group B (n = 70)	*P*-value
**Immediate PPB, no. (%)**	**6 (9.0)**	**22 (31.4)**	**0.001**
Grade 1, no. (%)	2 (3.0)	5 (7.1)	
Grade 2, no. (%)	1 (1.5)	3 (4.3)	
Grade 3, no. (%)	1 (1.5)	11 (15.7)	
Grade 4, no. (%)	2 (3.0)	3 (4.3)	
Immediate PPB grades 1–2, no. (%)	3 (4.5)	8 (11.4)	0.208
Immediate PPB grades 3–4, no. (%)	3 (4.5)	14 (20.0)	**0.008**
**Delayed PPB, no. (%)**	**5 (7.5)**	**5 (7.1)**	**0.943**
Minor bleeding, no. (%)	5 (7.5)	3 (4.3)	0.487
Significant bleeding, no. (%)	0 (0.0)	2 (2.9)	0.497
**Total bleeding events, no. (%)**	**11 (16.4)**	**25 (35.7)**	**0.010**

PPB, postpolypectomy bleeding

Delayed PPB occurred in 5 polyps in each group. In group A, all cases were minor bleeding that stopped spontaneously without an endoscopic procedure. In contrast, there were 2 cases of significant bleeding in group B, which occurred 5 and 24 h after the procedure, respectively, and were successfully treated with clip applications.

PPB, including immediate and delayed, occurred in 11 polyps (16.4%) in group A and in 25 polyps (35.7%) in group B, which was significant (*P* = 0.010). Two polyps in group B presented with both immediate and minor delayed PPB.

Although hospital stay was prolonged in four patients (2 patients in each group respectively) because of delayed PPB, overall mean duration of the hospital stay was not different between the groups (group A 1.5 ± 0.8 days vs. group B 1.6 ± 0.6 days, *P* = 0.433).

### Predictors associated with immediate PPB

To identify the predictors associated with immediate PPB, univariate analyses were performed (Table 3). The stalk diameter was classified by the median value (≥4 vs. 1–3 mm). Polyp size was categorized according to the previous studies (≥20 vs. 10–19 mm) [18–20]. Prophylactic clip application decreased the occurrence of immediate PPB (odds ratio [OR] 0.215, 95% confidence interval [CI] 0.081–0.571, *P* = 0.002). In addition, polyp size ≥20 mm and stalk diameter ≥ 4 mm increased the risk of immediate PPB compared with polyp size < 20 mm and stalk diameter < 4 mm. Polyps in the left-sided colon tended to have a higher risk of immediate PPB than those in the right-sided colon in the analysis (OR 2.207, 95% CI 1.063–5.913, *P* = 0.049). Other factors such as antiplatelet use and bowel preparation were not significant.

**Table 3:** Univariate analyses of predictors associated with immediate postpolypectomy bleeding

Variables	OR	95% CI	*P*-value
Age (≥60 vs. < 60)	1.187	0.515–2.734	0.687
Sex (male vs. female)	1.035	0.377–2.843	0.946
DM (yes vs. no)	1.333	0.336–5.290	0.682
HTN (yes vs. no)	0.708	0.285–1.757	0.456
BMI (≥24 vs. < 24)	1.826	0.789–4.228	0.160
Use of antiplatelet (yes vs. no)	0.607	0.165–2.226	0.451
Prophylactic clip application (yes vs. no)	**0.215**	**0.081**–**0.571**	**0.002**
Bowel preparation (excellent or good vs. adequate)	0.418	0.163–1.070	0.069
Polyp size (≥20 mm vs. 10–19 mm)	**3.504**	**1.482–8.287**	**0.004**
Stalk diameter (≥4 mm vs. 1–3 mm)	**2.507**	**1.063–5.913**	**0.036**
Location of polyp (left colon vs. right colon)	**2.677**	**1.005–7.130**	**0.049**
High-grade adenoma (yes vs. no)	0.482	0.104–2.244	0.352
Adenocarcinoma (yes vs. no)	1.600	0.294–8.716	0.587

OR, odds ratio; CI, confidence interval; HTN, hypertension; DM, diabetes mellitus; BMI, body mass index

Table 4 shows the predictors associated with total bleeding events including immediate and delayed PPB. Similar findings to those of immediate PPB (Table 3) alone were noted. Prophylactic clip application also decreased the bleeding events (OR 0.354, 95% CI 0.157–0.795, *P* = 0.012).

**Table 4:** Univariate analyses of predictors associated with total bleeding events (immediate and delayed postpolypectomy bleeding)

Variables	OR	95% CI	*P*-value
Age (≥60 vs. < 60)	0.925	0.428–2.000	0.844
Sex (male vs. female)	0.975	0.390–2.38	0.956
DM (yes vs. no)	0.929	0.237–3.642	0.916
HTN (yes vs. no)	0.655	0.284–1.509	0.320
BMI (≥24 vs. < 24)	1.295	0.604–2.777	0.506
Use of antiplatelet (yes vs. no)	0.857	0.289–2.536	0.780
Prophylactic clip application (yes vs. no)	**0.354**	**0.157**–**0.795**	**0.012**
Bowel preparation (excellent or good vs. adequate)	0.428	0.176–1.039	0.061
Polyp size (≥20 mm vs. 10–19 mm)	**3.790**	**1.698–8.459**	**0.001**
Stalk diameter (≥4 mm vs. 1–3 mm)	2.060	0.927–4.580	0.076
Location of polyp (left colon vs. right colon)	**2.702**	**1.122–6.505**	**0.027**
High-grade adenoma (yes vs. no)	0.846	0.257–2.785	0.783
Adenocarcinoma (yes vs. no)	1.129	0.209–6.095	0.887

OR, odds ratio; CI, confidence interval; HTN, hypertension; DM, diabetes mellitus; BMI, body mass index

## Discussion

In this study, we found that prophylactic clip application for large pedunculated polyps (size ≥1 cm) decreased the occurrence of immediate PPB, particularly cases of grade 3–4 bleeding that required endoscopic management. However, prophylactic clip application did not reduce the rate of delayed PPB.

Although prophylactic clip application reduced the occurrence of immediate PPB by > 20% in the present study, the rate of overall PPB (28 of 137, 20.4%) was still higher than that reported in previous studies. This finding may be attributable to the inclusion of large polyps (> 1 cm in diameter). Moreover, other studies on polypectomy of large and giant polyps reported bleeding rates equal to 12% [21] and even 24% [22], comparable to our results. In addition, other plausible explanations for the high PPB rate can be offered. First, more than one-third of the enrolled patients had hypertension. Second, the bleeding risk may increase with antiplatelet use, although patients discontinued antiplatelet therapy 5–7 days before the procedure. Third, our study included grade 1–2 immediate PPB that did not require endoscopic management. The inclusion of minor bleeding in the analysis might have caused a high incidence of PPB. Finally, in this study, electrocoagulation (Endocut mode) was used for reproducibility of the incision quality during all polypectomies, which automatically fractionates the cutting and coagulation phases [23].

To date, the prophylactic endoscopic strategies to prevent PPB include injection with epinephrine-saline [11], ablation with argon plasma coagulation [24], and use of mechanical devices such as a detachable snare [7, 19] or clips. A meta-analysis showed that both injection and mechanical therapies were superior to non-prophylactic therapy in preventing the occurrence of immediate PPB, although there were no statistically significant differences among prophylactic therapies [25]. The disadvantages of prophylactic management during colonoscopic polypectomy include the temporary effect of injection therapy, thermal tissue injury of ablation therapy, and technical difficulty of placing the detachable snare appropriately.

In this study, grade 3–4 immediate PPB occurred in 3 polyps despite prophylactic clip application. Of these 3 polyps in group A, 2 polyps had 5-mm-sized stalks, which have difficulty in applying optimal clip placement at the stalk because of its length and difficult position. Although prophylactic clip application could not completely prevent immediate PPB, this method has several advantages, as follows: (i) longer effect than that of submucosal injection, (ii) no thermal injury, (iii) easier installment on the stalk, and (iv) less expensive than a detachable snare. A pilot study on the efficacy of prophylactic clip application for pedunculated polyps demonstrated that the immediate PPB rate was 3.6% (2 of 56) and that clipping might be an effective technique [14]. However, the study did not have a comparator group.

A previous prospective randomized study investigating the usefulness of prophylactic clip application for pedunculated polyps ≥1 cm was terminated without reaching the target sample size because of the unexpectedly high rate (10.6%) of severe bleeding, mucosal burn, and perforation in the clip application group [15]. The authors suggested that the short pedicle of the polyp made it easier to apply a hemoclip; however, it led to mucosal burns and perforation. A comparison study between clip application alone and clip application plus injection of epinephrine-saline in pedunculated colon polyps showed immediate PPB rates of 12.0 and 14.4%, respectively (*P* = 0.64) [26]. Because the study did not include a comparison with a non-prophylactic group, it did not prove the usefulness of the prophylactic clipping method. As far as we know, our study is the first randomized controlled study to identify the benefit of prophylactic clip application in large pedunculated polyps.

However, prophylactic clip application did not prevent delayed PPB in the present study. The plausible explanation was as follows; First, prophylactic clips might not cover the stalk of large pedunculated polyps completely; second, insufficient sample size owing to the small number of delayed PPB events was likely to have contributed to the insignificant benefit. Our results corresponded with those of a Japanese study [27], recently published randomized trial [28], and systemic review [29] that reported that prophylactic clip application on the resected site after polypectomy did not decrease the rate of delayed PPB. In contrast, Liaquat et al. [20] reported that prophylactic clipping of polypectomy sites reduced the risk of delayed PPB in their retrospective study. The study included cases of clipping after endoscopic resection of large sessile and flat lesions; therefore, it is difficult to compare the result with that of our study.

Nevertheless, significant delayed bleeding was not occurred in group A while 2 cases of significant bleeding were occurred in group B. The prophylactic clips might be effective in preventing the significant delayed PPB although there was not statistical significance. The polyp size is known to be the most important predictor of PPB in colonoscopic polypectomy [10, 30, 31]. In addition, the stalk diameter is also a significant risk factor for PPB in large pedunculated polyps [32]. Generally, there are nourishing blood vessels in the stalk of the pedunculated polyps, and the size of blood vessels depends on the size of polyp and the diameter of stalk [29, 33]. Our study re-confirmed that polyp size ≥20 mm and stalk diameter ≥ 4 mm were significant predictors of immediate PPB compared with polyp size < 19 mm and stalk diameter < 3 mm. However, the efficacy of prophylactic clips in sessile polyps might be different.

The current study has several limitations. First, our enrollment target was 192 patients per arm. However, current study did not reach the target sample size because of the slow enrollment of patients and the unexpectedly high rate of PPB in the non-clipping group. Therefore, the study might be underpowered because of the small sample size. However, continuing this study despite the high occurrence of immediate PPB in group B could be unethical. Second, a single experienced endoscopist in a single center performed all procedures to minimize the effect of endoscopist- or procedure-related variables. However, consequently, the results might not be applicable to other endoscopists with different experience level or techniques. Third, we enrolled patients with underlying cardiovascular disease. Therefore, our results might not be generalizable to patients with chronic renal failure or chronic liver disease. Finally, our study did not compare the role of clip with other prophylactic measures like epinephrine injections as a third arm owing to the sample size.

## Conclusions

Prophylactic clip application may decrease the occurrence of immediate PPB in large pedunculated colorectal polyps ≥1 cm in size. However, the downside of prophylactic clipping for all patients with pedunculated polyps is that it might increase the cost and time of the procedures. Thus, a pedunculated polyp of size ≥2 cm and stalk diameter ≥ 4 mm may be a good indication for prophylactic clip application.

## Data Availability

The datasets used and/or analyzed during the current study are available from the corresponding author on reasonable request.

## Abbreviations

BMI: body mass index
CI: confidence interval
CRC: colorectal cancer
DM: diabetes mellitus
HTN: hypertension
INR: international normalized ratio
OR: odds ratios
PPB: postpolypectomy bleeding
PTT: partial thromboplastin time
SD: standard deviation
SPSS: Statistical Package for the Social Science
